# The benefit of tissue sealant on urethroplasty in hypospadias patients – A systematic review and meta-analysis

**DOI:** 10.12688/f1000research.108503.2

**Published:** 2024-01-30

**Authors:** Safendra Siregar, Steven Steven, Akhmad Mustafa

**Affiliations:** 1 Urology department, Hasan Sadikin Academic Medical Center, Universitas Padjadjaran, Bandung, West Java, 40161, Indonesia

**Keywords:** Hypospadias, Urethroplasty, Tissue Sealant, Benefit

## Abstract

**Background:**

Hypospadias was ranked second after undescended testis as the most prevalent congenital abnormality in newborn males. Hypospadias can be successfully repaired through multiple surgeries in the majority of children. Postoperative complications were not rarely seen after surgeries, such as urethrocutaneous fistula (UCF), meatal stenosis, and glans breakdown. Tissue sealant application in hypospadias repair serves as additional suture line coverage and reduces the post surgery complications. However, the effects of sealants usage during urethroplasty are still uncertain. This review aimed to know the effects of tissue sealant usage on patients with hypospadias who undergo urethroplasty.

**Methods:**

The study was reported based on the Preferred Reporting Items for Systematic Reviews and Meta-Analysis (PRISMA) guidelines. Literature was searched on PubMed, Embase, and Scopus following PRISMA guidelines. The search was conducted on October 12th, 2021, using the search term (“glue” OR “sealants” OR “tissue glue” OR “tissue sealant” OR “tissue adhesive”) AND (“hypospadias” OR “urethrocutaneous fistula” OR “urethral repair” OR “urethroplasty” OR “hypospadiology”).

**Result:**

Systematic searching from all databases resulted in 160 potential articles. After a full-text review, eight articles were included in this study. Urethrocytaneous fistula complication was reported in all studies. The occurrence of complication reported by all studies was urethrocutaneous fistula. Several studies also reported tissue edema and flap-related complications. Tissue sealant had no significant effect in reducing meatal stenosis.

**Conclusions:**

This systematic review revealed additional benefits from several types of tissue sealant in hypospadias repair surgery. Fibrin sealant application over the urethroplasty suture line in hypospadias repair offers a water-proof coverage and may enhance the outcome from the surgery.

## Introduction

Hypospadias was ranked second after undescended testis as the most prevalent congenital abnormality in newborn males. ^1^ Hypospadias is a displaced urethral opening at the penis ventral side because of incomplete penile structures closure during embryogenesis. ^1^At present, there are more than 300 known methods of surgical management of hypospadias however, hypospadias repair remains a complex procedure, even for sophisticated urologiest and paediatrics surgeons. ^2^

Nowadays, hypospadias can be successfully repaired through one-stage surgery in the majority of children. ^3^^,^^4^ Study by Arya et al., showed the overall success rate of one staged sugery was 73.5% that defined as no fistula, stricture, or residual chordee, and glandular meatus with acceptable cosmetic. The success rate after revision surgery reached 94.77%. ^5^ However, several study found that staged uretroplasty with or without grafting gave higher success rate compared to single staged procedure. ^6^^,^^7^

In the recent past, the role of tissue sealants has been taken into consideration. ^8^ Sealants are agents that can prevent the leakage of fluids by providing a physical barrier, which also aids in hemostasis. ^9^ Tissue sealant application in hypospadias repair serves as additional suture line coverage and reduces the post surgery complications. ^8^ However, the effects of sealants usage during urethroplasty are still uncertain. This review aimed to know the effects of tissue sealant usage on patients with hypospadias who undergo urethroplasty.

## Methods

### Eligibility criteria

Criteria for inclusion:

1. The study was published in full-text and written in English.
2. Published between 2002 and 2021.
3. Randomized controlled trials (RCTs) or prospective cohort studies.
4. Participants aged up to 18 years, who required operative repair for hypospadias.
5. The intervention included tissue sealants usage.
6. The comparison was made with patients without tissue sealant (Control).
7. The outcome of the studies was the number of children with complication after sealant use.

### Guidelines

**Figure 1.**
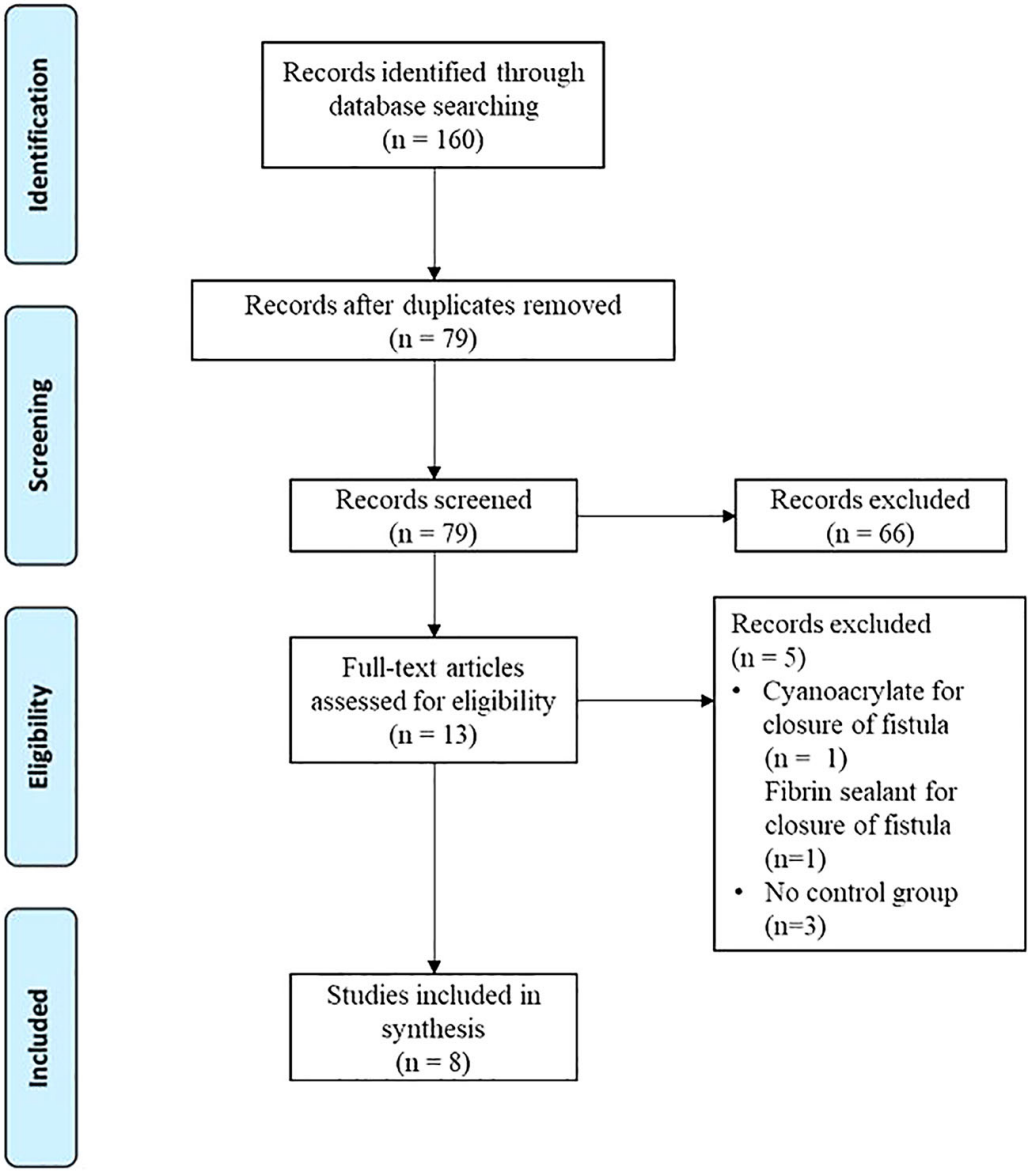
Study selection using PRISMA flow diagram.

The Preferred Reporting Items for Systematic Reviews and Meta-Analysis (PRISMA) guidelines was used in reporting this study. ^7^ The flow diagram can be found in Figure 1.

### Search strategy

The literature search was performed on PubMed, Embase, and Scopus following PRISMA guidelines (See reporting guidelines). The search was conducted on October 12th, 2021, using the search term (“glue” OR “sealants” OR “tissue glue” OR “tissue sealant” OR “tissue adhesive”) AND (“hypospadias” OR “urethrocutaneous fistula” OR “urethral repair” OR “urethroplasty” OR “hypospadiology”).

### Data extraction

One reviewer (SS) performed literature selection and data extraction to an Excel database. Titles and abstracts were screened by two reviewers (SFS and AM) to determine the qualified articles. Next, the full text was reviewed to gather detailed information. Data were extracted by two reviewers (DK and SS) independently. Authors, study design, publication year, population, sample size, age, hypospadias severity, type of procedure performed, and characteristics of sealant information were extracted to Microsoft Excel. Any discrepancies among the observers were discussed until consensus was reached.

## Result

### Study selection

Systematic searching from all databases resulted in 160 potential articles ( Figure 1). Duplicates were checked and excluded, leaving 79 articles. A total of 13 articles were qualified in this study. After a full-text review, eight articles were selected for review. These studies used various tissue sealant types. All the studies reported the procedure.

### Study characteristics and key findings

The general characteristics of reviewed articles are listed in Table 1. All studies are prospective in study design. A total of 863 subjects with hypospadias were included. They were divided into the sealant and non-sealant groups, with 494 and 369 participants.

**Table 1.** Studies characteristics.

Study number	Author	Study design	Mean age	Sample size		Sealant type	The volume of sealant and technique
				Sealant group	No sealant group		
1	Shenoy *et al.*, 2021	Prospective	S-3.8 years N-4.6 years	20	20	Fibrin sealant	Application of fibrin sealant on the neo-urethra's suture line and no vascular cover was used
2	Hosseinpour *et al.*, 2019	Prospective	-	300	100	Cryocalcium glue	5 ml of cryoprecipitate and 1 ml of calcium gluconate; thin layer on the urethral and dartos suture lines
3	Guinot *et al.*, 2014	Prospective	S- median 8 months	33	72	Autologous PRF	The PRF was applied over the urethroplasty. A few interrupted suture with resorbable polyglyconate 7/0 was performed to secure the line.
4	Kocherov *et al.*, 2014	Prospective	S-32.1 months N-26.7 months	20	20	BioGlue	A thin layer was formed on the neo-urethra's second suture line.
5	Hosseini *et al.*, 2012	Prospective	13.5 months	20	41	Cyanoacrylate	Cyanoacrylate was poured over the glans. A rubbery consistency formed in 60 seconds, then reapplied 4-6 times.
6	Kajbafzadeh *et al.*, 2011	Prospective	S-12.18 years	11	-	Fibrin glue	A fibrin glue layer over the closure area was applied, and the dartos fascia layer was brought over it. Another layer was placed and a skin closure was performed.
7	Gopal *et al.*, 2008	RCT	S-28.02 months N-28.00 months	60	60	Fibrin glue	A thin layer in the urethral and dartos suture line
8	Ambriz-González *et al.*, 2007	Prospective	S-33.5 months N-31.3 months	30	56	Fibrin glue	2 ml of fibrin glue over the surgical site and suture line

Shenoy *et al.*, ^11^ Gopal *et al.*, ^12^ and Ambriz-González *et al*., ^13^ used fibrin sealant in their study. There were several distinctions regarding the application of fibrin sealant. Shenoy *et al*. ^11^ applied the sealant on the neo-urethra's suture line, and no vascular cover was used. Then, Gopal *et al*. ^12^ made a thin layer of the sealant in the urethral and dartos suture line. Whereas Ambriz Gonzalez *et al*. ^13^ applied 2 ml of fibrin glue over the surgical site and suture line.

In another study, Guinot *et al*. ^14^ applied autologous platelet-rich fibrin (PRF) over urethroplasty. Meanwhile, Kajbafzadeh *et al*. ^15^ aimed to determine fibrin sealant efficacy for UCF repair after multiple hypospadias and epispadias surgery. Over the closure area, a fibrin glue cover was made, and the dartos fascia layer was placed over this area.

Other sealants used in the included study were cryocalcium glue, BioGlue, and cyanoacrylate. Hosseinpour *et al*. ^16^ used cryocalcium glue as a thin urethral layer and dartos suture lines. Kocherov *et al*. ^17^ applied BioGlue as a thin cover on the the neo-urethra second suture line. Lastly, Hosseini *et al*. ^18^ poured cyanoacrylate over the glans. A rubbery consistency was formed in 60 seconds, then reapplied 4-6 times.

Key findings of reviewed articles are listed in Table 2. Postoperative follow-up ranges between 7 days to 2 weeks to evaluate immediate complications, and between 1 month to 5 years to evaluate late complications. Hypospadias location differs from coronal, subcoronal, distal penile, etc. Some studies reported statistically significant differences of complication between the sealant and non-sealant groups. A study by Shenoy *et al*. reported distinction in early postoperative ooze, skin flap-related complications, and tension between the sealant and non-sealant groups (p<0.05). ^11^ Hosseinpour *et al*. revealed differences between edema and fistula (p=0.002). ^16^ Kocherov *et al*. reported differences in poor cosmetic outcomes (p=0.007). ^17^

**Table 2.** Main findings.

Study number	Author	Time to follow up	Hypospadias location/type		Main finding		Other findings
			Sealant group	Non-sealant group	Sealant group	Non-sealant group	
1	Shenoy *et al.*, 2021	7 days after discharge, 1, 3, and 6 months	Coronal (1), subcoronal (9), distal penile (4), mid penile (4), proximal penile (1), penoscrotal (1)	Coronal (4), subcoronal (5), distal penile (1), mid-penile (7), proximal penile (1), and penoscrotal (2)	• Complications in 5 patients • Complications: coronal fistula (3), poor cosmetic outcome (3)	• Complications in 9 patients • Complications: early postoperative ooze (2), skin flap-related complications (3), UCF (7), poor cosmetic outcome (7), penile torsion (5)	• Differences in early postoperative ooze, skin flap-related complications and torsion were significant (p<0.05) • There were fewer patients with UCF and poor cosmetics in the fibrin sealant group • The overall improvement in outcome was found to be significantly different among the two groups, with a higher number in group 1 (p<0.05)
2	Hosseinpour *et al.*, 2019	2 weeks of the procedure, then every 3 month	Distal penile (300)	Distal penile (100)	• Complications: edema (24), fistula (5), meatal stenosis (10)	• Complications: edema (15), fistula (6). Diverticulum (1), meatal stenosis (4)	• Statistically significant differences in edema (p=0.02) • Statistically significant differences in fistula (p=0.02) • No case of allergic in the case group
3	Guinot *et al.*, 2014	2 month and 6 months post-operative	Distal (33)	Distal (72)	• Coronal fistula (2)	• Urethral fistula (3)	• No statistically significant difference between both groups (p=0.65)
4	Kocherov *et al.*, 2014	15 ± 2.3 months after surgery (mean ± SD)	Distal shaft (7), mid-shaft (6), proximal shaft (1), peno-scrotal (6)	Distal shaft (8), mid-shaft (5), proximal shaft (1), peno-scrotal (6)	• Complications: UCF (4), breakdown of the suture line (4), meatal stenosis (1), poor cosmetic (n=12.6%)	• Complications: UCF (3), breakdown of the suture line (1), meatal stenosis (1), poor cosmetic (n=19.95%)	• Statistically significant differences in poor cosmetic (p=0.007)
5	Hosseini *et al.*, 2012	2 weeks postoperative and six to twelve months	Type B and C hypospadias	Type B and C hypospadias	• Complications: hematoma (1), skin necrosis (1)	• Complications: hematomas (7), painful removal of the dressing (12), repeat dressing (10)	-
6	Kajbafzadeh *et al.*, 2011	First 3 months from the date of surgery	Proximal hypospadias (8), epispadias (1), bladder exstrophy complex (1)	-	• Wound dehiscence (1), subcutaneous hematoma (1)	-	• A patient with the largest fistula (7 mm) had partial wound dehiscence.
7	Gopal *et al.*, 2008	2 weeks of the procedure and then every 3 months for the 1 ^st^ year, then every 6 months for next 5 year	-	-	• Complications in 12 patients • Complications: postoperative edema (10), fistula (6), infection (4), meatal stenosis (3), proximal stricture (3).	• Complications in 34 patients • Complications: postoperative edema (21), fistula (19), diverticulum (3), infection (2), meatal stenosis (3), proximal stricture (10)	• Statistically significant differences in postoperative edema (p=0.039), fistula formation (p=0.027), and overall complication (p=0.003)
8	Ambriz-González *et al.*, 2007	At least 6 months after surgery	Proximal shaft (8), medial shaft (7), distal shaft (15)	Proximal shaft (14), medial shaft (12), distal shaft (30)	• Complications: urethrocutaneous fistula (3), flap dehiscence (4), flap necrosis (2)	Complications: urethrocutaneous fistula (23), flap dehiscence (28), flap necrosis (16)	• Statistically significant differences in urethrocutaneous fistula (p=0.002), flap dehiscence (p=0.001), and flap necrosis (p=0.01)

Shenoy *et al*. ^11^ and Kajbafzadeh *et al*. ^15^ reported less complication in the fibrin glue than in the non-sealant group. In the non-sealant group, the complications included early postoperative ooze, skin flap-related complications, UCF, poor cosmetic outcome, penile torsion, postoperative edema, diverticulum, meatal stenosis, proximal stricture, flap dehiscence, and flap necrosis. Early postoperative ooze, skin flap-related complications, and torsion were significantly different in the sealant and non-sealant groups (p<0.05). However, in a study by Kajbafzadeh *et al*., ^15^ the difference of complications between both groups was not statistically significant.

Hosseinpour *et al*., ^16^ in their study about the application of Cryocalcium glue, revealed that there was no allergy found in the sealant group. Then, Kocherov *et al*. ^17^ reported poorer cosmetic outcomes in the non-sealant group compared to the BioGlue group (p=0.007). Subsequently, Hosseini *et al*. ^18^ found poorer results in the non-sealant group than the cyanoacrylate group. His study revealed some complications, including hematomas, painful dressing removal, and repeat dressing.

In addition, Ambriz-González *et al*. compared the use of fibrin sealant on proximal, medial, and distal hypospadias. ^13^ No statistically significant differences were found in the use of fibrin glue in proximal hypospadias to prevent complications. For medial hypospadias, fibrin glue was found to significantly reduce the occurrence of urethrocutaneus fistula (p=0.03) and flap dehiscence (p=0.001). Then, for distal hypospadias, fibrin glue was also found to reduce the occurrence of urethrocutaneus fistula (p=0.04) and flap dehiscence (p=0.04). ^13^

**Figure 2.**
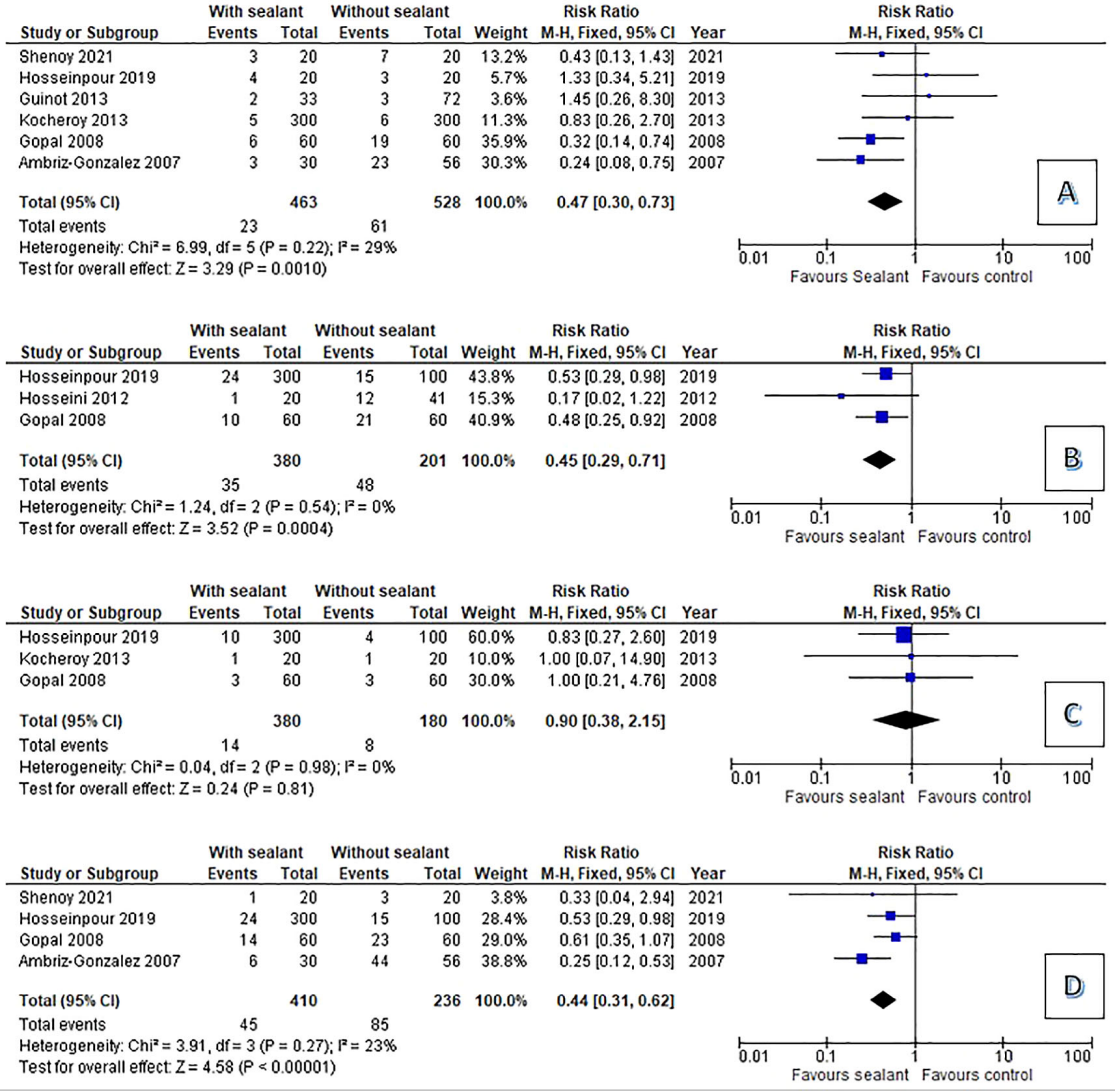
Forest plot: the occurrence of complication after urethroplasty (A: urethrocutanous fistula, B: tissue edema, C: meatal stenosis, D: Flap related complication).

The analysis of clinical and statistical findings were shown in Figure 2. We compared the occurance of complication after hypospadias repair. The occurrence of UCF was reported in all studies. UCF with sealant complication was the occurrence of complication reported by all studies was urethrocutaneous fistula. The pool risk ration (RR) for UCF was 0.47 (95% CI 0.30 – 0.73 p=0.001) compared to non-sealant group. Tissue edema and flap related complication also reported by several studies with pooled RR for tissue edema and flap related complication was 0.45 (95% CI0.29 – 0.71 p=0.0004) and 0.44 (95% CI 0.31 – 0.62 p<0.0001). Tissue sealant gave no significant effect in reducing meatal stenosis with Pool RR 0.9 (95% CI 0.38 – 2.15 p=0.8).

## Discussion

Hypospadias is a congenital anomaly discovered in 1 of every 300 live births. ^13^ About 2-15% of infants with hypospadias experienced complications following repair procedures. ^19^ The most common complications found, in order, are fistula, stricture, and dan wound breakdown. ^19^ The need for hypospadias repairing technique, especially for cosmetic issues, is very demanding. The most popular technique for distal hypospadias surgery is 'tabularized incised plate urethroplasty’ or TIP and it is reported that the complication is as high as 5.5% with most common complication was UCF 0–10%. ^20^ Various tissue sealants were offered to gain a better seal of neo-urethra formation in the urologic procedures. ^17^

This systematic review revealed five different sealant types were used in hypospadias repair. The sealants are classified into natural and synthetic. The natural sealants are made of albumin, fibrin, and cryocalcium, while the synthetic sealant is made of cyanoacrylate.

### Fibrin glue

Fibrin glue contains fibrinogen-rich coagulation cascade, factor XII, dan thrombin. ^8^ According to the coagulation cascade, scavenging macrophages will reabsorb the fibrin glue in 14 days so that it has a weak mechanical strength. ^5^ Other complications are anaphylactic reaction and infection. ^21^

Fibrin sealant has been used in many surgical procedures for hemostasis (topical agent) and tissue approximation (as an adhesive). Its usage has expanded through numerous procedures, including urologic surgery. Urologic procedures need the fibrin glue's sealing power to improve wound healing in penile urethroplasty. ^22^

Tisseel, a fibrin glue preparation, was first utilized by Kinahan *et al*. to enhance hypospadias repair. ^23^ In addition, Hick *et al*. stated that fibrin glue could be beneficial in promoting early catheter removal and accelerating the process of wound healing. ^24^ Study from Barbagli *et al*. shared their experience using fibrin glue in the buccal mucosa graft urethroplasty for bulbar urethral stricture. ^25^

Better postoperative outcomes were found in patients with fibrin sealant usage during surgery. There were no flap necrosis or penile torsion found as early postoperative complications. ^11^ Havez *et al*. stated this benefit might be influenced by the regeneration of the cellular and angiogenic of the tunica albuginea defect. ^26^ Urethra-cutaneous fistula was found less in the sealant group. They also had a better cosmetic outcome. There is a statistically significant improvement in the overall outcomes. ^27^

Another complication assessed in the study was postoperative edema. A larger number of early postoperative edema incidences in the non-sealant group might be due to undiscovered microscopic leakage in the tissue spaces between the suture lines. This could lead to a fistula formation. ^13^ The application of fibrin glue forms an additional layer over the urethral tube, seals minute cracks between sutures, and decreases post-surgery edema. Therefore, its application might accelerate the wound healing process. ^13^

Commercial fibrin sealants had a higher risk of allergic reactions compared to the homologous, blood bank product, fibrin sealants, which are prepared from single unit plasma. Risk of infection could be decreased by using autologous blood as the fibrinogen source. The setback is that the production took longer and created non-uniform fibrinogen concentration. ^28^^,^^29^ Kajbafzadeh *et al*. described fibrin glue's benefits preventing further recurrence in patients with recurrent fistula after hypospadias or epispadias repair. ^15^

### Autologous platelet-rich fibrin

Dohan *et al*. ^30^ developed platelet-rich fibrin (PRF) in the early 2000s that has been used to augment the process of tissue healing. The procedure is simple and less expensive than fibrin glue: the PRF patch is collected by centrifuging the autologous blood sample without any adjuvant. The patch creation could be done amid the urethroplasty procedure and used promptly. ^14^^,^^31^^,^^32^

A study by Guinot *et al*. ^14^ found no negative effect of PRF as there were no adverse effects or skin infections discovered. Unfortunately, PRF superiority over conventional covering technique has not been proven.

### Cryo-calcium glue

The use of cryo-calcium glue has been limited in underdeveloped or developing countries. This is due to its higher risk of infection transmission than fibrin glue, despite having been through donor screening, heat-treating, and the use of solvent and detergent suspension. ^16^ The preparation of cyrocalcium glue is very simple and can be done during the urethroplasty procedure. It can be used shortly after its preparation. ^16^ It is formed from combining a patient's cryoprecipitate and calcium gluconate. ^16^

This cryo-calcium glue could be a safe and less expensive alternative than commercial fibrin glue to reduce fistula formation. The case group showed a minimal incidence of fistula by using cryo-calcium glue. ^16^ It is also safe from allergic complications due to its autologous origin. ^16^

### BioGlue

In a prospective study, Kocherov *et al*. reported the efficacy of BioGlue in hypospadias repair. ^17^ BioGlue does not carry a risk of infection transmissions, such as HIV, HBV, or HCV, different from natural human fibrin tissue sealant. Therefore, it appears to be an ideal sealant. ^33^ BioGlue consists of two purified bovine serum albumin and glutaraldehyde. ^34^ Glutaraldehyde acts to bridge the bovine serum albumin amine groups to the target tissue's extracellular matrix, forming a covalent bond between tissue and adhesive. ^33^ However, the advantages of BioGlue in reducing fistula formation and reconstruction breakdowns were not shown in the Kocherov *et al*. study. ^17^ No difference in fistula formation and surgical breakdown were seen in both groups. ^17^ In addition, the BioGlue group tends to have a more prominent severe fibrotic skin reaction, therefore inferior in cosmetics than the control group. Other publicity stated some discouraged effects of BioGlue, such as toxicity to tissue, local inflammation, and postoperative wound complications. ^34^^,^^35^ It is hypothesized due to glutaraldehyde toxicity. ^17^

### Cyanoacrylate

In a study by Hosseini *et al*., cyanoacrylate was used as a dressing in urethroplasty. ^18^ This dressing has many purposes with others in edema restriction, hematoma formation, and wound stabilization if treated in several layers and lacks urine and feces permeability. ^18^ There were no regular changes or pain upon removal. This type of dressing was more convenient for patients and the wound was kept hygienic at home. The occurrence of complications is less, 10% versus 30%; but late complications indirectly related to the dressing have been excluded. ^18^

Prestipino *et al*. used cyanoacrylate in four patients with UCF. Three of them had fistula healing. ^36^ In another study, cyanoacrylate was used for simple sharp wounds in various locations, including the face and extremities, inguinal and umbilical hernia, cleft lip, hypospadias, and post-hypospadias fistula repair. The results showed improvement in efficacy, cosmetics, procedure duration, and patient comfort. ^37^

### Tissue sealant effect

Based on our meta-analysis, tissue sealant can reduce urethroplasty complications during hypospadias repair. In our analysis, tissue sealant can reduce the occurrence of fistula urethocutan, tissue edema, and flap-related complication. This result is in line with the previous systematic review. ^5^

## Limitation

There are several limitations to this study. The included studies reported non-uniform complications. Information on the sealant and surgery cost of hypospadias repairment was limited. Moreover, several hypospadias types, various repair techniques used, and operator experience might serve as confounding factors as not all studies report on the complication and success rate based on the type of hypospadias.

## Conclusion

This systematic review revealed additional benefits from several types of tissue sealant in hypospadias repair surgery. Fibrin sealant applied over the urethroplasty suture lines forms an adequate water-proof cover and improves outcomes in hypospadias repair. Fibrin glue reduces the incidence of complications in medial and distal hypospadias but not in proximal hypospadias. The application of PRF had no harmful effects, but it did not demonstrate superiority over conventional covering techniques. Cryocalcium glue could be used as an alternative to fibrin glue to reduce fistula formation. It is also safe and less expensive than fibrin glue. The use of cyanoacrylate dressing lessens the immediate complication of hypospadias repair surgery. Meanwhile, BioGlue does not show any additional advantages in hypospadias repair in pediatric patients than the standard procedures. A conclusion cannot be made due to studies diversity. Therefore, randomized controlled trials with a significant sample size are necessary to precisely compare the sealant and non-sealant groups, types of sealants, and hypospadias location.

## Data availability

All data used in the research are available as part of articles and no additional source are needed to disclose.

## Reporting guidelines

Figshare: PRISMA checklist for ‘The Benefit of Tissue Sealant on Urethroplasty in Hypospadias Patients – A Systematic Review and Meta-analysis’, DOI: 10.6084/m9.figshare.19102184.

Data are available under the terms of the Attribution 4.0 International license (CC BY 4.0)

## Author contributions

**SFS:** Conceptualization, Methodology, Project administration, Supervision

**SS:** Data curation, Software, Writing original draft

**AM:** Conceptualization, Supervision, Validation, Wiriting review and editing
