# Supplementary material for: The benefit of tissue sealant on urethroplasty in hypospadias patients – A systematic review and meta-analysis
Source: F1000Res. 2024 Jan 30;11:184. Originally published 2022 Feb 14. [Version 2] doi: 10.12688/f1000research.108503.2 (PMC11226945; doi:10.12688/f1000research.108503.2)
Supplement: Peer review history

# Reviewer report: Reviewer response for version 2

**DOI:** 10.5256/f1000research.160687.r285077

**Published:** 2024-07-05

**Authors:** shilpa sharma

**Affiliations:** 1 All India Institute of Medical Sciences, New Delhi, India

1. The title states “The benefit of tissue sealant… However, the conclusion does not justify the title. I would suggest removing the first three words and beginning the title with “Tissue…
2. Introduction: The first two paragraphs (para 1 and 2) are well-known facts and do not add anything new to the manuscript. The Introduction can start with paragraph 3.
3. More details, mechanism of action and concerns with the use of sealants such as fibrosis can be mentioned.
4. It is not understandable how meatal stenosis may be prevented by the application of a sealant. Rather, a sealant may be a cause of meatal stenosis by causing fibrosis. In the current study, the result may be interpreted as “application of sealants did not increase the incidence of meatal stenosis”. The statement “Tissue sealant gave no significant effect in reducing meatal stenosis with Pool RR 0.9 (95% CI 0.38 – 2.15 p=0.8)” may be modified accordingly
5. A table on the type of sealants and their composition will add value to the manuscript with a comparison of costs involved.
6. Minor grammatical errors may be corrected eg. Spelling of urethrocutaneous in abstract and Medial should be middle hypospadias.

The discussion can include more recent articles on the topic [1,2].

Are the rationale for, and objectives of, the Systematic Review clearly stated?

Yes

Is the statistical analysis and its interpretation appropriate?

Yes

If this is a Living Systematic Review, is the ‘living’ method appropriate and is the search schedule clearly defined and justified? (‘Living Systematic Review’ or a variation of this term should be included in the title.)

Not applicable

Are sufficient details of the methods and analysis provided to allow replication by others?

Partly

Are the conclusions drawn adequately supported by the results presented in the review?

Yes

Reviewer Expertise:

pediatric urology, pediatric study, pediatric hepatobiliary surgery, pediatric colorectal surgery

I confirm that I have read this submission and believe that I have an appropriate level of expertise to confirm that it is of an acceptable scientific standard, however I have significant reservations, as outlined above.

## References

[1] : Looking beyond the dartos fascia and tunica vaginalis: reviewing the stance of common adjuvant covering biomaterials in hypospadiology. *Am J Clin Exp Urol* .2022;10(2) :111-118 35528465 PMC9077145

[2] : Use of an Autologous Platelet-Rich Concentrate in Hypospadias Repair: A Systematic Review and Meta analysis. *Arab J Urol* .2023;21(3) : 10.1080/2090598X.2022.2149129 177-184 10.1080/2090598X.2022.2149129 37521453 PMC10373602

# Reviewer report: Reviewer response for version 2

**DOI:** 10.5256/f1000research.160687.r242337

**Published:** 2024-02-06

**Authors:** Borko Stojanovic

**Affiliations:** 1 Belgrade Centre for Urogenital Reconstructive Surgery, Belgrade, Serbia; 2 School of Medicine, University of Belgrade, Belgrade, Serbia

The authors have made a substantial improvement of the manuscript. This is a very important topic, and this systematic review/meta analysis will be beneficial in hypospadias surgery and literature.

Are the rationale for, and objectives of, the Systematic Review clearly stated?

Partly

Is the statistical analysis and its interpretation appropriate?

Partly

Are sufficient details of the methods and analysis provided to allow replication by others?

Yes

Are the conclusions drawn adequately supported by the results presented in the review?

Partly

Reviewer Expertise:

Paediatric urology

I confirm that I have read this submission and believe that I have an appropriate level of expertise to confirm that it is of an acceptable scientific standard.

# Reviewer report: Reviewer response for version 1

**DOI:** 10.5256/f1000research.119884.r185863

**Published:** 2023-09-07

**Authors:** V V S Chandrasekharam

**Affiliations:** 1 Ankura Hospitals for Women and Children, Hyderabad, Telengana, India

This is a systematic review & meta-analysis (SR & MA) on an interesting topic. However, a similar review as published recently ^1^, which found no difference in the overall complication rate with the use of tissue sealants. The present article by Siregar et al has several drawbacks, some of which the authors themselves have observed and mentioned. Since an SR & MA represents a high level of evidence, it is important to apply certain stringent criteria to define the quality of the included studies. 

1. In the methodology of SR, it is important to calculate the heterogeneity of the included papers by the use of I ^2 ^statistics and Funnel plot. The publication bias of each included paper needs to be assessed and reported using methods such as Doi plot and LFK index. These statistics provide the reader with information regarding the comparability of the included papers and thus is a measure of the reliability of the results of the SR & MA. Without these data, it is impossible for the reader to arrive at any conclusions.  
2. The authors themselves have acknowledged that the included studies have several shortcomings in their methodology, reporting of complications, type of sealant used and follow-up. This makes the results of the SR & MA unreliable.
3. In the discussion, the authors should compare their results with other similar studies ^1^ and explain the differences in the outcomes.
4. The single most important criteria for the success of hypospadias repair is the expertise and experience of the surgeon. This factor has not been discussed in the studies not in the SR & MA.

Thus, although this SR & MA is a good effort, the results must be interpreted with caution.

Are the rationale for, and objectives of, the Systematic Review clearly stated?

Yes

Is the statistical analysis and its interpretation appropriate?

Partly

Are sufficient details of the methods and analysis provided to allow replication by others?

Partly

Are the conclusions drawn adequately supported by the results presented in the review?

No

Reviewer Expertise:

PEDIATRIC UROLOGY & Hypospadias

I confirm that I have read this submission and believe that I have an appropriate level of expertise to confirm that it is of an acceptable scientific standard, however I have significant reservations, as outlined above.

## References

[1] : Can sealing promote healing?: A systematic review and meta-analysis highlighting the adjunctive role of tissue sealant application during urethroplasty for hypospadias. *J Pediatr Urol* .2021;17(6) : 10.1016/j.jpurol.2021.07.011 805-812 10.1016/j.jpurol.2021.07.011 34417129

# Reviewer report: Reviewer response for version 1

**DOI:** 10.5256/f1000research.119884.r123542

**Published:** 2022-02-28

**Authors:** Borko Stojanovic

**Affiliations:** 1 Belgrade Centre for Urogenital Reconstructive Surgery, Belgrade, Serbia; 2 School of Medicine, University of Belgrade, Belgrade, Serbia

This systematic review deals with a very interesting and up-to-date topic, since successful hypospadias repair still presents a great challenge. Literature data on the use of tissue sealant in urethroplasty show quite heterogeneous results, and authors made a very good choice to assess this by a systematic review. The methodology is based on PRISMA guidelines, with detailed explanation on search strategy, as well as good illustration of results. However, I have to note that the rest of the manuscript is poorly written, in several points:

1. The manuscript should be copy-edited to improve the quality of the writing.
2. Rationale and objectives are poorly explained, and introduction section needs more details and corrections, especially about the use of tissue sealant in hypospadias repair. Is it indicated in every case and form? What type of tissue sealant and when...? 
  Introduction Section: The sentence " *Hypospadias can be successfully repaired through multiple surgeries in the majority of children*" has to be corrected. Today, most of hypospadias cases are repaired by a one-stage surgery. 
  Introduction Section: " *The overall success rate for first surgery was 55.9%.*". Please provide reference. The rate of success is much higher today. 
  Abstract Section: " *UCF complication was reported in all studies.*" Do not start a sentence with an acronym.
3. Some sentences within the Results and Discussion sections are unclear and require copy-editing.
  Section Discussion: " *Although superior in cosmetic issues, it is reported that the complication is as high as 20-30%.* ^*17*" ^I don't think this data is true, based on provided reference.
4. Diversity of the studies is large, and it should be primarily denoted in Conclusion section. 

Are the rationale for, and objectives of, the Systematic Review clearly stated?

Partly

Is the statistical analysis and its interpretation appropriate?

Partly

Are sufficient details of the methods and analysis provided to allow replication by others?

Yes

Are the conclusions drawn adequately supported by the results presented in the review?

Partly

Reviewer Expertise:

Paediatric urology

I confirm that I have read this submission and believe that I have an appropriate level of expertise to confirm that it is of an acceptable scientific standard, however I have significant reservations, as outlined above.

# Response

**Published:** 2023-12-27

**Authors:** Steven Steven

**Affiliations:** Padjadjaran University, Indonesia, Indonesia

Thank you for the review. We tried to revise the article in line with the recommendation you made. we also update several data according to newer articles.
